# Correlates of HIV Testing Among Men Who have Sex with Men in Three Urban Areas of Mozambique: Missed Opportunities for Prevention

**DOI:** 10.1007/s10461-015-1044-8

**Published:** 2015-05-19

**Authors:** Roberta Z. Horth, Beverly Cummings, Peter W. Young, Joy Mirjahangir, Isabel Sathane, Rassul Nalá, Tim Lane, H. Fisher Raymond

**Affiliations:** 1 Global Health Sciences, University of California, San Francisco, CA USA; 2 Tulane University School of Public Health and Tropical Medicine, New Orleans, LA USA; 3 Division of Global HIV/AIDS, Centers for Disease Control and Prevention (CDC), Maputo, Mozambique; 4 San Francisco Department of Public Health, San Francisco, CA USA; 5 International Training and Education Center for Health (I-TECH), Maputo, Mozambique; 6 Instituto Nacional de Saúde, Ministry of Health, Maputo, Mozambique

**Keywords:** HIV testing, Men who have sex with men (MSM), Respondent-driven sampling (RDS), Mozambique, Africa

## Abstract

This is the first study to identify levels of recent HIV testing and associated factors among men who have sex with men (MSM) in Mozambique. Using data from Maputo (n = 493), Beira (n = 572), and Nampula/Nacala (n = 347), collected via respondent-driven sampling in 2011, and excluding those with prior known infection, we found that 30.4 % [95 % confidence interval (CI) 25.0–36.3 %], 42.1 % (95 % CI 36.8–47.3 %) and 29.8 % (95 % CI 22.9–36.9 %), respectively, had recently tested for HIV (≤12 months), while between three and five out of 10 MSM had never tested. A range of factors was associated with recent HIV testing such as familiarity with the modes of transmission, knowledge of antiretroviral treatment for HIV, contact with peer educators and awareness of partner serostatus; yet, surprisingly recent healthcare utilization was not associated with recent testing. Findings provide evidence that structural and behavioral interventions among MSM may play an important role in increasing HIV testing.

**Electronic supplementary material:**

The online version of this article (doi:10.1007/s10461-015-1044-8) contains supplementary material, which is available to authorized users.

## Resumen

Este es el primer estudio realizado entre hombres que tienen sexo con hombres (HSH) en Mozambique que identifica los niveles de la prueba reciente del VIH y los factores asociados. Se recogió datos de tres sitios, Maputo (n = 493), Beira (n = 572), y Nampula/Nacala (n = 347), en 2011 a través de un muestreo dirigido por los entrevistados, se excluyo los participantes que tenían conocimiento de su seroestado de HIV positivo, y se encontró que el 30.4 % (Intervalo de Confianza [IC] de 95 %: 25.0-36.3 %), 42.1 % (IC 95 %: 36.8-47.3 %) y 29.8 % (IC 95 %: 22.9-36.9 %) de los HSH, respectivamente, había hecho la prueba de VIH recientemente (≤ 12 meses), mientras que entre tres y cinco en cada 10 HSH nunca había la hecho. Se encontró una serie de factores que se asocia con pruebas recientes de VIH, tales como el conocimiento de los modos de transmisión, el conocimiento de tratamiento con antirretrovirales, el contacto con los educadores de pares y el conocimiento del estado serológico de una pareja, pero sorprendentemente la utilización reciente de servicios de salud no se asoció con la prueba reciente. Los resultados proporcionan evidencia de que las intervenciones estructuráis y del comportamiento pueden desempeñar un papel importante en el aumento de la razón de las pruebas del HIV.

## Introduction

Increasing knowledge of HIV status among key populations at higher risk of infection, such as men who have sex with men (MSM), is key to reducing new HIV infections [1]. HIV testing, as an intervention itself, underlies the effectiveness of most other prevention approaches [2] and has been identified as a priority for HIV prevention by the World Health Organization (WHO) [3]. HIV testing enables undiagnosed HIV positive persons to become aware of their infection, gain access to early treatment services and receive counseling on transmission prevention behaviors [1].

The rates of HIV testing around the world historically have been low, even in areas with high prevalence of HIV. According to estimates from 32 sub-Saharan African (SSA) countries, the proportion of the male adult population receiving an HIV test and obtaining test results in the last 12 months ranged from 1.6 % in Niger to 41.7 % in Eritrea. Mozambique’s estimate falls in the middle of the range at 9.0 % based on the last National Aids Indicator Survey, although the country ranks amongst the highest in terms of prevalence of HIV with 11.5 % of adults aged 15–49 infected [4].

In SSA, including Mozambique, there is limited information on the extent of recent HIV testing and knowledge of HIV serostatus among MSM. A 2009 meta-analysis of lifetime HIV testing rates for MSM found that less than a third of MSM on average had ever received an HIV test [5]; yet, those estimates included only two SSA countries (Ghana and Nigeria, with rates of 25.2 and 30.2 %, respectively). Since then, multiple studies among MSM in SSA have been published presenting a diverse range of lifetime HIV testing figures, including 19.2 % in Zanzibar, Tanzania [6], 35.2 % in Blantyre and Lilonge, Malawi [7], 37.9 % in Soweto, South Africa [8], 38.2 % in Luanda, Angola [9], 59.4 % in Windhoek, Namibia [7], 69.3 % in Ehlanzeni district, Mpumalanga Province, South Africa (~a 2 h drive from Maputo) [10], 81.6 % in Douala and Yaoundé, Cameroon [11], and 82.9 % in Gaborone, Botswana [7].

The Mozambican government, having prioritized the national expansion of HIV voluntary counseling and testing services for the general population, has experienced rapid expansion of counseling and testing services across the country [12]. However, key populations have not specifically been prioritized as part of this expansion and it is not known to what extent testing services are reaching these populations. The high prevalence of HIV in key populations at higher risk for HIV in Mozambique point to the urgent need to develop effective HIV prevention strategies that include the expansion of HIV testing [13, 14]. While some studies in SSA have looked at factors associated with HIV testing among MSM [11, 15, 16], this is the first study to identify levels of recent HIV testing and associated factors among MSM in Mozambique.

Current approaches to HIV prevention being implemented include biomedical and behavioral interventions among individuals who are aware of their HIV-positive status, such as ‘test and treat’ [17] and ‘positive health, dignity and prevention (PHDP)’ [18]. Yet, the success of such interventions require high levels of HIV testing [19]. This analysis provides evidence that donors and policy makers can use in making decisions about adopting such strategies among key populations in Mozambique. By understanding the characteristics of MSM who recently tested for HIV, programs can prioritize individuals in need of HIV testing and adequately address barriers to achieving universal HIV testing.

## Methodology

### Sampling and Study Population

Cross-sectional surveys using respondent-driven sampling (RDS) were conducted in the cities of Maputo, Beira, and Nampula/Nacala. Enrollment took place between June and December of 2011. Survey cities were selected based on their population size and geographic representativeness of each region of Mozambique (North, Central, and South). Surveys were conducted at discreet locations rented specifically for the duration of the survey. Additional details have been published elsewhere [20, 21].

Participants were men at least 18 years old who had had sex (oral or anal) with a man in the 12 months preceding the survey. Additionally, participants must have possessed a valid referral coupon given to them by a member of their social network, could not have previously participated in the survey, and had worked, resided or socialized in the one of the survey areas for at least 6 months prior to participating. The term socialized was used to capture participants who did not live in the city yet came into the city to meet friends in their MSM network. Participants received coupons specific to each city and could only participate in the survey in the city from which their coupon originated.

Participation in the survey was anonymous. Participants were asked to provide consent in writing. All participants wanting to know their serostatus were offered an HIV rapid test on-site. Those testing positive were given referrals to a nearby public healthcare facility trained in providing services for MSM. The study was approved by the National Bioethics Committee for Health (*Comité Nacional de Bioética para a Saúde*) of Mozambique, the University of California at San Francisco, and by the Center for Global Health of the US Centers for Disease Control and Prevention.

### Measures

Behavioral data were collected using a standardized survey tool developed from questionnaires used in other countries [22]. The survey included questions related to demographic characteristics, sexual risk behaviors, HIV knowledge and utilization of health services, including HIV testing services. The survey was developed in English and Portuguese. It was programmed electronically using Questionnaire Development System (QDS™) version 2.6.1 and administered by interviewers using a netbook computer.

The primary outcome of interest of this analysis is recent HIV testing (defined as having been tested for HIV by self-report within the 12 months prior to participation in the study). Participants were asked detailed questions concerning their sexual experiences with their last five sexual partners (male or female) in the past 12 months. These questions gathered information about partner’s gender, age, number and types of sexual acts, condom use during sexual acts, and knowledge of partner’s HIV status. Participants were also asked if they had been involved in any transactional sex (defined as having given or received goods, money or services in exchange for sex).

### Laboratory Testing

Blood specimens were collected from participants through either a finger stick or venous blood draw, and dried blood spots (DBS) were prepared on filter paper. These specimens were linked to behavioral data using an alphanumeric laboratory code. The samples were tested for HIV using sequential testing at the serology laboratory of the National Institute of Health (INS). Screening was performed using Vironostika HIV Uniform II plus O (bioMérieux SA, France). Reactive samples and 5 % of negative samples were confirmed with Murex HIV 1.2.O (Murex Biotech Ltd, UK). Discrepant results were retested using Genscreen HIV 1/2 Version 2 (Bio-Rad, France).

### Data Analysis

Data were cleaned and analyzed using R version 2.15 (R Development Core Team, 2011). Since RDS methodology was not designed for aggregation across survey sites, data for each survey site were analyzed independently. This analysis excludes participants who reported having ever tested positive for HIV prior to participating in the survey and those who did not answer questions about whether or when they had ever been tested for HIV.

Population estimates and bootstrapped confidence intervals (Table 1) were produced in RDSAT version 7.1 (www.respondentdrivensampling.org) using the RDS-II estimator. Network size outliers above 5 % were pulled in and the algorithm type was set to “enhanced data-smoothing”. RDSAT-adjusted data are presented for all variables; the only exception being in the analysis of self-reported reasons for having or not having tested for HIV (Table 4) as these samples are small and were not designed to make inferences about the MSM population of each city.

**Table 1:** RDS-weighted characteristics of MSM in Maputo, Beira, and Nampula/Nacala, Mozambique 2011

Variables	Maputo			Beira			Nampula/Nacala		
	Crude		Adjusted^a^	Crude		Adjusted^a^	Crude		Adjusted^a^
	n/N	%	% (95 % CI)	n/N	%	% (95 % CI)	n/N	%	% (95 % CI)
Demographic characteristics									
Ages 18–25^b^	383/493	77.7	82.8 (76.8–87.8)	450/572	78.7	80.5 (75.6–84.6)	289/347	83.3	83.3 (77.0–89.1)
Completed primary school	421/493	85.4	80.5 (74.8–85.5)	520/572	90.9	91.2 (88.2–94.1)	239/347	68.9	66.3 (58.5–73.5)
Away from primary residence for >1 month in past 12 months	158/493	32.0	27.5 (22.2–32.8)	155/572	27.1	24.1 (19.2–28.7)	124/347	35.7	31.4 (24.6–37.6)
HIV testing and health seeking behaviors									
Knew where to go to get an HIV test	462/492	93.9	94.1 (91.7–96.4)	546/571	95.6	94.2 (91.1–96.5)	319/347	91.9	89.5 (85.4–94.0)
Never had an HIV test	207/493	42.0	47.7 (42.2–53.6)	195/572	34.1	36.7 (31.4–42.3)	161/347	46.4	57.4 (49.1–64.4)
Self-reported to have tested for HIV in the past 12 months	170/493	34.5	30.4 (25.0–36.3)	256/572	44.8	42.1 (36.8–47.3)	132/347	38.0	29.8 (22.9–36.9)
Had a moderate or high perceived risk of having HIV	196/493	39.8	39.9 (34.2–45.5)	124/572	21.7	20.2 (15.8–24.5)	102/347	29.4	27.5 (21.5–33.1)
Had contact with a peer educator in the past 12 months	214/491	43.6	38.2 (32.0–44.2)	140/570	24.6	22.2 (17.9–27.2)	175/347	50.4	40.8 (33.2–47.5)
Used a healthcare service in the past 12 months	246/493	49.9	48.0 (42.2–54.3)	205/572	35.8	37.2 (32.6–42.1)	236/347	68.0	68.0 (61.8–74.1)
HIV knowledge									
Did not know that condoms can protect against HIV	39/493	7.9	9.2 (6.0–12.7)	32/572	5.6	5.8 (3.5–8.5)	23/347	6.6	5.9 (3.1–9.6)
Did not know that a healthy-looking person can have HIV	24/493	4.9	6.0 (3.3–9.0)	21/572	3.7	4.9 (2.7–7.4)	47/347	13.5	16.4 (11.2–22.4)
Thought that a person can get HIV by sharing a meal with someone who is infected	46/493	9.3	10.2 (6.8–14.0)	34/572	5.9	8.3 (5.3–11.6)	16/347	4.6	5.6 (2.6–8.7)
Had heard about ARV drugs	420/493	85.2	81.0 (75.4–85.9)	521/572	91.1	90.9 (87.8–93.6)	318/347	91.6	90.1 (85.3–93.9)
HIV and STI’s									
Has an undiagnosed HIV infection	47/444	10.6	7.5 (4.1–11.8)	47/571	8.2	7.6 (4.8–10.4)	10/345	2.9	4.4 (2.1–7.7)
Had a self-reported genital sore or ulcer in the past 12 months	30/493	6.1	4.3 (2.2–6.3)	68/572	11.9	10.9 (7.7–13.9)	20/347	5.8	2.8 (0.8–5.4)
Sexual behaviors									
Had >5 sexual partners in the past 12 months	84/493	17.0	13.8 (9.8–18.0)	63/572	11.0	7.1 (4.7–9.5)	25/347	7.2	5.0 (2.6–8.1)
Had VAI with a female partner in the past 12 months	331/493	67.1	73.5 (67.1–79.1)	251/572	43.9	42.7 (35.9–48.7)	205/347	59.1	63.8 (56.3–71.0)
Had transactional AI with a male partner in the past 12 months	246/492	50.0	46.9 (39.9–53.1)	209/571	36.6	38.7 (33.6–44.5)	175/347	50.4	43.3 (35.6–50.4)
Had transactional VAI with a female partner in the past 12 months	116/493	23.5	23.7 (18.8–28.8)	67/571	11.7	12.1 (8.5–16.1)	87/347	25.1	24.6 (18.9–31.1)
Sexual behaviors with last partners (up to five) in the past 12 months									
Had any unprotected insertive AI with a male partner	153/493	31.0	28.9 (23.5–34.7)	142/572	24.8	32.0 (27.2–37.0)	164/347	47.3	44.5 (37.7–51.1)
Had any unprotected receptive AI with a male partner	61/493	12.4	8.5 (5.6–11.8)	57/572	10.0	9.9 (6.8–13.1)	94/347	27.1	24.7 (18.9–31.1)
Had any unprotected VAI with a female partner	209/493	42.4	48.3 (41.5–54.5)	78/572	13.6	13.3 (9.8–17.1)	147/347	42.4	47.6 (40.0–55.0)
Knew the HIV status of at least one partner	189/493	38.3	33.1 (27.6–38.6)	194/572	33.9	28.1 (23.5–32.5)	136/347	39.2	33.9 (28.7–40.5)

*AI* anal intercourse, *ARV* antiretroviral, *STI* sexually transmitted infection, *VAI* vaginal or anal intercourse

^a^Point prevalence and boot-strapped confidence intervals produced using the RDS-II estimator in RDSAT 7

^b^Mean age 22.3 in Maputo, 22.0 in Beira and 21.9 in Nampula/Nacala

Individualized weights produced in RDSAT were imported into R for weighted logistic regression (Tables 2, 3) to measure associations between independent variables and recent HIV testing. The Health Belief Model was used as the theoretical framework for variable selection and modeling, particularly looking at variables that fit into the model’s four main constructs of perceived susceptibility, severity, barriers and benefits [23]. Predictor variables independently associated at a level of *p* ≤ 0.2 in bivariate analysis, and those that were considered essential elements of the model or possible confounders were included in multivariate analysis. Variance inflation factors were computed for independent variables to identify collinearity problems in multivariate modeling. ANOVA was used to compare the change in variance between a full model and models with reduced set of variables to assess the contribution of each variable. Wald test was used to test the contribution of individual regression coefficients in the final model.

**Table 2:** RDS-weighted bivariate analysis of factors associated with recent HIV testing (<12 months) in Maputo, Beira, and Nampula/Nacala, Mozambique, 2011

Variables	Maputo					Beira						Nampula/Nacala					
	Crude	Adjusted				Crude		Adjusted				Crude		Adjusted			
	n/N	% Recently HIV tested (95 % CI)^a^	OR (95 % CI)^b^	z-score^c^	*p* value^c^	n/N		% recently HIV tested (95 % CI)^a^	OR (95 % CI)^b^	z-score^c^	*p* value^c^	n/N		% recently HIV tested (95 % CI)^a^	OR (95 % CI)^b^	z-score^c^	*p* value^c^
*Perceived susceptibility*																	
Had a moderate or high perceived risk of having HIV																	
No	106/297	31.3 (24.7–38.3)	1.00			208/448		44.3 (38.5–50.3)	1.00			103/245		34.8 (26.7–43.0)	1.00		
Yes	64/196	28.8 (20.9–38.2)	0.8 (0.5–1.1)	−1.26	0.21	48/124		33.1 (23.0–43.7)	0.6 (0.4–1.0)	−2.11	0.04	29/102		17.4 (8.9–28.5)	0.4 (0.2–0.7)	−2.99	0.003
Knew the HIV status of at least one partner in the past 12 months																	
No	83/304	26.8 (20.4–33.6)	1.00			147/378		36.8 (30.2–42.8)	1.00			69/211		25.2 (17.4–32.9)	1.00		
Yes	87/189	38.1 (28.9–48.2)	1.8 (1.2–2.7)	2.92	0.003	109/194		55.3 (45.8–63.9)	2.0 (1.4–2.9)	3.74	<0.001	63/136		37.8 (27.4–49.3)	1.5 (0.9–2.4)	1.60	0.11
Had unprotected AI with a male partner in the past 12 months																	
No	117/314	32.5 (25.7–39.4)	1.00			195/418		45.1 (38.1–50.9)	1.00			68/158		35.2 (24.5–43.9)	1.00		
Yes	53/179	25.4 (17.5–34.9)	0.7 (0.5–1.1)	−1.51	0.13	61/154		34.7 (26.0–43.5)	0.6 (0.4–0.8)	−2.85	0.004	64/189		25.7 (17.6–34.3)	0.6 (0.4–1.0)	−1.97	0.048
Had unprotected VAI with a female partner in the past 12 months																	
No	99/284	30.4 (23.1–38.6)	1.00			222/494		43.1 (37.4–48.6)	1.00			85/200		37.6 (28.0–46.6)	1.00		
Yes	71/209	30.3 (22.7–38.6)	0.9 (0.6–1.3)	−0.66	0.51	34/78		35.4 (20.8–49.6)	0.8 (0.5–1.3)	−0.97	0.33	47/147		21.5 (13.7–31.2)	0.5 (0.3–0.8)	−2.87	0.004
Had transactional AI with a male partner in the past 12 months																	
No	77/246	26.4 (18.9–34.3)	1.00			164/362		40.8 (33.6–47.0)	1.00			51/172		23.9 (15.8–33.2)	1.00		
Yes	92/246	35.3 (27.6–43.3)	1.5 (1.0–2.2)	2.11	0.04	92/209		44.6 (36.0–53.2)	1.2 (0.9–1.7)	1.12	0.26	81/175		38.1 (27.2–47.9)	1.9 (1.2–3.1)	2.80	0.005
Had transactional VAI with a female partner in the past 12 months																	
No	129/377	31.6 (24.9–38.3)	1.00			231/504		42.7 (36.8–48.1)	1.00			97/260		29.9 (21.7–37.5)	1.00		
Yes	41/116	27.5 (18.3–37.5)	0.7 (0.4–1.1)	−1.49	0.14	24/67		37.1 (23.0–51.5)	0.7 (0.4–1.2)	−1.17	0.24	35/87		29.3 (18.6–43.1)	0.8 (0.4–1.3)	−0.94	0.35
*Perceived severity*																	
Did not know that a healthy-looking person can have HIV																	
No	160/469	29.2 (23.8–35.1)	1.00			249/551		42.2 (36.8–47.6)	1.00			116/300		28.8 (22.3–37.3)	1.00		
Yes	10/24	50.2 (23.2–75.3)	2.4 (1.2–5.0)	2.41	0.02	7/21		40.3 (13.3–65.7)	1.0 (0.4–2.2)	−0.01	0.99	16/47		30.3 (14.5–48.3)	1.1 (0.6–2.0)	0.28	0.78
Thought someone can get HIV by sharing a meal with someone																	
No	162/447	32.3 (26.5–38.9)	1.00			248/538		43.5 (37.9–48.9)	1.00			124/331		28.4 (21.4–35.5)	1.00		
Yes	8/46	13.4 (2.4–28.2)	0.3 (0.1–0.7)	−2.68	0.007	8/34		25.8 (7.6–46.3)	0.4 (0.2–0.8)	−2.62	0.009	8/16		55.2 (23.8–81.1)	2.7 (1.0–7.3)	1.98	0.048
*Perceived benefits*																	
Had heard about ARV drugs																	
No	12/73	16.8 (6.9–28.0)	1.00			16/51		32.9 (17.8–49.1)	1.00			6/29		6.9 (0.0–16.4)	1.00		
Yes	158/420	33.3 (27.0–39.4)	2.5 (1.4–4.8)	3.08	0.002	240/521		43.0 (37.3–48.4)	1.5 (0.8–2.9)	1.31	0.19	126/318		31.4 (23.9–38.5)	4.4 (1.5–18.9)	2.41	0.02
*Perceived barriers*																	
Knew where to go to get an HIV test																	
No	1/30	9.0 (0.0–23.3)	1.00			2/25		9.5 (0.0–26.7)	1.00			2/28		6.8 (0.0–15.2)	1.00		
Yes	169/462	31.5 (25.2–38.1)	6.0 (1.7–41.1)	2.35	0.02	254/546		43.5 (37.5–49.2)	7.7 (2.7–31.1)	3.40	0.001	130/319		32.2 (23.7–39.3)	11.7 (3.2–94.8)	3.03	0.002
Spoke Portuguese as primary language at home																	
No	28/108	24.4 (13.7–36.7)	1.00			100/235		41.6 (33.1–48.8)	1.00			39/102		25.5 (17.2–37.0)	1.00		
Yes	142/385	32.3 (25.8–38.9)	1.6 (1.0–2.6)	1.80	0.07	156/337		42.6 (35.4–49.3)	1.1 (0.8–1.5)	0.29	0.77	93/245		32.2 (23.5–39.8)	1.5 (0.9–2.5)	1.40	0.16
*Cues to action*																	
Had a self-reported genital sore or ulcer in the past 12 months																	
No	160/463	30.9 (25.2–36.8)	1.00			226/504		42.5 (36.4–47.5)	1.00			125/327		30.1 (23.0–37.0)	1.00		
Yes	10/30	21.1 (8.3–41.4)	0.5 (0.1–1.5)	−1.08	0.28	30/68		37.1 (22.5–52.0)	0.8 (0.5–1.4)	−0.82	0.41	7/20		15.0 (3.5–36.6)	0.4 (0.1–1.3)	−1.34	0.18
Used a healthcare service in the past 12 months																	
No	77/247	28.3 (20.9–35.3)	1.00			162/367		42.7 (35.5–48.6)	1.00			38/111		31.9 (20.5–44.2)	1.00		
Yes	93/246	32.7 (24.4–41.2)	1.1 (0.7–1.6)	0.31	0.76	94/205		41.1 (32.7–49.3)	0.9 (0.6–1.2)	−0.83	0.41	94/236		28.5 (20.4–35.9)	0.9 (0.5–1.4)	−0.51	0.61
Had contact with a peer educator in the past 12 months																	
No	74/277	26.7 (19.5–33.3)	1.00			177/430		39.6 (33.4–44.9)	1.00			57/172		25.2 (17.6–34.3)	1.00		
Yes	95/214	37.5 (28.5–46.6)	1.5 (1.0–2.2)	1.91	0.06	77/140		49.8 (38.2–60.9)	1.5 (1.0–2.2)	2.14	0.03	75/175		35.8 (26.1–44.9)	1.5 (0.9–2.4)	1.68	0.09
*Demographic and sociopsychological factors*																	
Ages 18–25 years																	
No	43/110	29.9 (23.3–36.6)	1.00			57/122		49.9 (36.1–59.5)	1.00			28/58		27.0 (11.4–41.9)	1.00		
Yes	127/383	32.1 (19.8–44.5)	0.9 (0.6–1.4)	−0.44	0.66	199/450		39.3 (33.0–45.7)	0.7 (0.5–1.1)	−1.69	0.09	104/289		28.7 (21.8–37.2)	0.9 (0.5–1.6)	−0.37	0.71
Completed primary school																	
No	13/72	21.5 (10.0–33.8)	1.00			13/52		28.7 (11.3–44.6)	1.00			35/108		23.1 (13.6–36.7)	1.00		
Yes	157/421	32.9 (26.8–39.4)	2.1 (1.3–3.7)	2.84	0.004	243/520		43.2 (37.4–48.4)	1.5 (0.8–2.8)	1.24	0.21	97/239		32.8 (23.7–40.1)	1.3 (0.8–2.1)	0.91	0.36
Away from primary residence >1 month in the past 12 months																	
No	112/335	31.3 (24.5–37.8)	1.00			191/417		43.4 (36.5–49.6)	1.00			71/223		26.0 (18.1–33.8)	1.00		
Yes	58/158	28.8 (19.6–39.8)	1.0 (0.6–1.5)	1.46	0.99	65/155		38.1 (29.1–47.1)	0.8 (0.5–1.1)	−1.28	0.20	61/124		37.7 (25.7–49.8)	1.8 (1.1–3.0)	2.46	0.01

*AI* anal intercourse, *ARV* antiretroviral, *STI* sexually transmitted infection, *VAI* vaginal or anal intercourse

^a^Point-estimates and boot-strapped confidence intervals produced using the RDS-II estimator in RDSAT 7

^b^ *OR* odds ratios, produced using individualized weights exported from RDSAT 7

^c^z-score and *p* value based on Wald-test of weighted logistic regression using RDSAT-produced individualized weights

**Table 3:** RDS-weighted multivariate analysis of factors associated with recent HIV testing (<12 months) in Maputo, Beira, and Nampula/Nacala, Mozambique, 2011

Variables	Maputo (N = 488)			Beira (N = 568)			Nampula/Nacala (N = 346)		
	AOR^a^ (95 % CI)	z-score^b^	*p* value^b^	AOR^a^ (95 % CI)	z-score^b^	*p* value^b^	AOR^a^ (95 % CI)	z-score^b^	*p* value^b^
Perceived susceptibility									
Had a moderate or high perceived risk of having HIV	0.83 (0.53–1.29)	−0.82	0.41	0.63 (0.39–1.00)	−1.95	0.052	0.46 (0.24–0.87)	−2.33	0.02
Had transactional anal intercourse with a male partner in the past 12 months	2.09 (1.35–3.27)	3.28	0.001	1.19 (0.81–1.73)	0.89	0.37	2.06 (1.24–3.46)	2.78	0.006
Had transactional vaginal or anal intercourse with a female partner in the past 12 months	0.51 (0.28–0.87)	−2.39	0.02	0.75 (0.41–1.35)	−0.94	0.35	0.78 (0.41–1.45)	−0.77	0.44
Knew the HIV status of at least one partner in the past 12 months	1.51 (0.97–2.35)	1.82	0.07	2.00 (1.36–2.96)	3.49	<0.001	1.47 (0.85–2.53)	1.38	0.17
Perceived severity									
Did not know that a healthy-looking person can have HIV	4.25 (1.84–10.17)	3.35	0.001	1.24 (0.51–2.97)	0.49	0.63	1.41 (0.68–2.87)	0.95	0.35
Thought that a person can get HIV by sharing a meal with someone who is infected	0.37 (0.13–0.87)	−2.13	0.03	0.38 (0.18–0.75)	−2.69	0.007	2.84 (0.96–8.77)	1.87	0.06
Perceived benefit									
Had heard about antiretroviral drugs	1.95 (1.00–4.00)	1.90	0.06	1.16 (0.58–2.39)	0.42	0.68	3.96 (1.22–17.96)	2.07	0.04
Perceived barrier									
Knew where to go to get an HIV test	3.97 (1.07–27.56)	1.76	0.08	8.01 (2.76–33.3)	3.38	0.001	8.76 (2.26–72.40)	2.61	0.009
Cues to action									
Had contact with a peer educator in the past 12 months	1.83 (1.19–2.84)	2.72	0.007	1.49 (0.98–2.26)	1.88	0.06	1.10 (0.65–1.86)	0.35	0.726
Demographic and sociopsychological factors									
Age (per year)	1.03 (0.98–1.08)	1.35	0.18	1.0 (0.96–1.05)	0.05	0.96	1.07 (1.00–1.14)	1.89	0.06
Completed primary school	1.96 (1.10–3.64)	2.22	0.03	0.75 (0.36–1.62)	−0.74	0.46	1.26 (0.72–2.26)	0.80	0.43
Away from primary residence >1 month in the past 12 months	0.90 (0.56–1.44)	−0.44	0.66	0.62 (0.40–0.94)	−2.20	0.03	1.88 (1.09–3.24)	2.28	0.02

^a^ *AOR* adjusted odds ratio, adjusted for all other variables in the model

^b^z-score and* px* value based on Wald-test of weighted logistic regression using RDSAT-produced individualized weights

## Results

### Demographic and Behavioral Characteristics

The study enrolled 496 participants (six seeds) in Maputo, 583 (three seeds) in Beira, and 353 (eight seeds) in Nampula/Nacala. Recruitment took place over 18 weeks in Maputo and Beira and 22 weeks in Nampula/Nacala. The maximum number of recruitment waves was 15 in Maputo and 23 in Beira and Nampula/Nacala. Participants with any HIV-positive test result prior to the survey and those who did not know if or when they had ever been tested for HIV were excluded resulting in a sample of 493 MSM in Maputo (99 % of original sample), 572 in Beira (98 %), and 347 in Nampula/Nacala (98 %). The measure of homophily for the outcome variable, recent HIV testing, was 0.163 in Maputo, 0.087 in Beira, and 0.283 in Nampula/Nacala. Median network size for participants in those same cities respectively was 10, 7 and 7.

Most participants were under the age of 25 in each location, and mean age was 22 across sites (Table 1). In those same cities, respectively, 80.5, 91.2 and 66.3 % of MSM had completed primary school and about one in four MSM (27.5, 24.1 and 31.4 %) had been away from their primary residence for a period of over 1 month in the past 12 months.

The majority of MSM (94.1, 94.2 and 89.5 %) knew where to go to get an HIV test; however, 47.7, 36.7 and 57.4 % had never been tested. Between four and seven out of every 10 MSM (48.0, 37.2 and 68.0 %) had used a healthcare service and between two and four out of every 10 MSM (38.2, 22.2 and 40.8 %) had contact with a peer educator in the 12 months preceding the survey in Maputo, Beira and Nampula/Nacala, respectively. The percentage of MSM that reported having an HIV test in the 12 months preceding the survey was 30.4, 42.1, and 29.8 %. Further, 7.5, 7.6 and 4.4 % of MSM had an undiagnosed HIV infection in Maputo, Beira and Nampula/Nacala, and 4.3, 10.9 and 2.8 % in those same cities had a self-reported genital sore or ulcer in the past 12 months.

Among MSM in Maputo, Beira and Nampula/Nacala, 46.9, 38.7 and 43.3 % had transactional sex with a male in the past 12 months, and 8.5, 9.9 and 24.7 % had unprotected receptive anal sex with a man in that same period. Sexual relations with women were also reported, with 73.5, 42.7 and 63.8 % of MSM having had anal or vaginal sex with a woman in the past 12 months and 23.7, 12.1 and 24.6 % having had transactional sex with a woman in that same period. About one-third (33.1, 28.1 and 33.9 %) knew the HIV status of at least one sex partner (male or female) in the past 12 months.

### Correlates of Recent HIV Testing

Recent testing was lower among MSM who perceived themselves to have a moderate or high risk of having HIV than among MSM who perceived themselves to have low or no risk of having HIV (33.1 vs. 44.3 % *p* = 0.04 in Beira and 17.4 vs. 34.8 %, *p* = 0.003 in Nampula/Nacala) (Table 2). Similarly, MSM in Beira and Nampula/Nacala who had unprotected anal sex with a male partner in the past 12 months had a lower percentage of recent testing than those who had protected sex (34.7 vs. 45.1 %, *p* = 0.004 and 25.7 vs. 35.2 %, *p* = 0.048, respectively), and the same was true for MSM in Nampula/Nacala who had unprotected anal or vaginal sex with a female partner in the past 12 months (21.5 vs. 37.6 %, *p* = 0.004). However, recent testing was higher among MSM in Maputo and Nampula/Nacala who had transactional sex with a male partner in the past 12 months than those who had not had transactional sex (35.3 vs. 26.4 %, *p* = 0.04 and 38.1 vs. 23.9 %, *p* = 0.005, respectively).

MSM in Beira who knew the HIV status of at least one sexual partner in the past 12 months had twice the odds [adjusted odds ratio (AOR) 2.00, 95 % confidence interval (CI) 1.36–2.96] of having recently tested for HIV compared with those who did not know the status of any partner (Table 3). MSM in Maputo and Beira who thought that a person can get HIV by sharing a meal with someone who has HIV were about a third less likely to have recently tested for HIV (AOR 0.37, 95 % CI 0.13–0.87, and AOR 0.38, 95 % CI 0.18–0.75, respectively) compared to those who knew correctly that a person cannot get HIV by sharing a meal with someone who has HIV. In Nampula/Nacala, MSM who had heard of antiretroviral drugs (ARV) were more likely to have tested recently (AOR 3.96, 95 % CI 1.22–17.96) compared to those who had never heard of ARV drugs. MSM in Beira and Nampula/Nacala who knew where to go to get an HIV test had eight times the odds (AOR 8.01, 95 % CI 2.76–33.3, and AOR 8.76, 95 % CI 2.26–72.40, respectively) of having tested recently compared with those who did not know where to test. In Maputo, MSM who had contact with a peer educator were more likely to have recently tested (AOR 1.83, 95 % CI 1.19–2.84) compared to those who had not had contact with a peer educator.

### Reasons for Never Having Tested or for Having Tested Recently

Among survey participants who never tested for HIV (n = 194, 185 and 159 in Maputo, Beira and Nampula/Nacala respectively), the main reasons given for not having done so was fear of discovering that they are HIV-positive (41.8, 26.5 and 34.6 % respectively) (Table 4). The next most common reason given was feeling they were not prepared for the test (10.3, 24.3 and 30.8 % respectively). The belief that they were not infected was reported as a reason by 8.2 % of participants in Maputo, 23.2 % in Beira, and 18.9 % in Nampula/Nacala. Not knowing where to go to have an HIV test was also given as a reason by 1.5, 13.5 and 6.3 % of participants in each city.

**Table 4:** Barriers and facilitators of HIV testing among survey participants (unadjusted data), Mozambique 2011

	Maputo		Beira		Nampula	
	n	%^a^	n	%^a^	n	%^a^
Reasons for never having an HIV test among participants that never had an HIV test before the survey^b^	n = 194		n = 185		n = 159	
Fear to discover that I am positive	81	41.8	49	26.5	55	34.6
I am not ready to get the test^c^	20	10.3	45	24.3	49	30.8
I am not infected	16	8.2	43	23.2	30	18.9
Don’t know where to go	3	1.5	25	13.5	10	6.3
Reasons for having an HIV test among participants who had a test in the 12 months preceding the survey^b^	n = 170		n = 256		n = 132	
Wanted to know my HIV status	78	45.9	172	67.2	120	90.9
I felt sick	22	12.9	6	2.3	0	0.0
My partner asked me to get tested	8	4.7	8	3.1	3	2.3
Advised by a health worker	8	4.7	13	5.1	0	0.0

^a^Estimates are not adjusted

^b^Multiple-choice question

^c^In Mozambique, HIV testing is generally done in the presence of the person; therefore, we assume that those who responded they were not prepared to test were also not prepared to receive results

Among participants who had tested for HIV in the 12 months preceding the survey (n = 170, 256 and 132 in each city respectively), most mentioned that they had tested because they wanted to know their status (45.9 % in Maputo, 67.2 % in Beira, and 90.9 % in Nampula/Nacala). In Maputo, 12.9 % of participants had tested for HIV because they felt sick, and in Beira and Maputo about five percent had tested because they had been advised to do so by a healthcare worker.

## Discussion

The aim of this study was to determine the levels and predictors of recent HIV testing among undiagnosed MSM in three urban areas of Mozambique. We found that only about one-third of MSM in the cities of Maputo, Beira and Nampula/Nacala had tested for HIV in the last 12 months, and that about three to five in every 10 MSM had never tested. These percentages are higher than those found among the general population of adult men in Mozambique [4] and higher than the global weighted average (31 % ever tested) [5]; however, they are below those of MSM in the neighboring country of Swaziland (50.7 % having tested in past year) [7] and fall far from the goal of universal annual testing, as recommended for populations with ongoing increased risk of HIV infection [24]. Additionally, these testing percentages bring to light an important gap in HIV prevention among MSM in Mozambique, where between 4 and 7 % of MSM have an undiagnosed HIV infection [20, 21].

Our findings also point to other gaps in prevention among MSM, such as the lack of knowledge of the modes of HIV transmission and unawareness of the availability of treatment for HIV, with at least one in 10 MSM not knowing about the existence of ARV, and about as many not knowing that a healthy-looking person can have HIV or thinking that HIV can be transmitted by sharing a meal. Consistent with other studies in Southern Africa [15, 25, 26], we found that knowledge about HIV is positively associated with testing behavior. Those who knew, correctly, that a person with HIV cannot transmit it by sharing a meal were more likely to test in all three cities. Also, congruent with the Health Belief Model, knowing that there are concrete benefits from HIV testing (e.g. access to life-extending treatment) can motivate MSM to test for HIV in Mozambique. These findings warrant an increase in HIV/AIDS educational programs among the MSM community in-country. However, the ability to increase such programming may be undermined by the government’s failure to recognize the country’s sole LGBT non-governmental organization, LAMBDA (The Mozambican Association for the Defense of Sexual Minorities) [27].

The lack of recognized MSM organizations in-country makes interpersonal relations an important factor to consider in HIV testing. Similar to other studies [11, 15, 16], we found that interpersonal relations may play an important role in testing behavior among MSM in Mozambique, as evidenced by the finding that those who knew the HIV status of a recent partner or those who had been in contact with a peer educator were more likely to have recently tested for HIV. Despite this, just three in 10 MSM knew the serostatus of a recent partner and only four in 10 MSM had been in contact with a peer educator in the past 12 months. Evidence-based interventions, that use social networks and community engagement [28, 29], including peer and small group education [30], may help increase HIV testing uptake among MSM and address barriers to serostatus disclosure between partners [31].

We believed that use of a healthcare service, as a cue-to-action in the Health Belief Model, would also be linked to increased HIV testing uptake; yet, surprisingly, we found no difference in recent HIV testing between those who had utilized a healthcare service in the past 12 months and those that had not. Among every 10 MSM that had used a healthcare service in the past 12 months, as many as six had not tested for HIV. Likewise, <5 % of MSM who had tested in the past 12 months reported having done so on advice by a healthcare worker. This result highlights the fact that key opportunities for testing MSM are being missed. These opportunities are likely being missed because MSM may be reluctant to disclose their sex practices to healthcare workers for fear of being stigmatized and discriminated against. As such, healthcare workers may not be aware of the prevalence of same sex behaviors in their communities or of the risks that such behaviors carry.

Direct and indirect stigma and discrimination in the health-care setting has been widely documented amongst MSM in Southern Africa [32–34] and Mozambique [35] given the lack of MSM-friendly services available in the region. The dual-stigma of being MSM and HIV-positive further exacerbates this issue. Structural interventions within the healthcare system to increase culturally-competent care, such as mentoring and training of providers, could help close the HIV testing gap [36]. Additionally, alternatives to provider-initiated testing, such as home-based testing, which has shown to be effective in increasing HIV testing uptake among MSM [37, 38], should be investigated as an alternative to facility-based HIV testing among MSM in Mozambique.

Of particular concern in the proliferation of HIV among the MSM community was the finding that MSM were less likely to have tested if they had potential recent exposure to HIV or a high self-perceived risk of having HIV. While we believe this is largely due to fear of receiving an HIV-positive result (at least a quarter of participants reported this fear as a reason for never having tested for HIV); further study is needed on the underlying reasons for this fear and to steps that can be taken to mitigate it. Early HIV infection plays a pivotal role in HIV transmission [39] and early diagnosis and intervention among MSM may be critical for reducing incidence of new infections in Mozambique.

We recognize several limitations of our study that are inherent in behavioral cross-sectional surveys. First, no causal effect can be drawn between predictor factors and HIV testing. Second, results are prone to self-reporting, self-selection, recall and social desirability bias. Lastly, being that the aim of the original survey was to determine HIV prevalence and associated risks; we were unable to include certain measures known to be associated with HIV testing, such as stigma. Similarly, the survey was not originally powered to detect associations with HIV testing; therefore, true associations could be masked by lack of power.

Despite these limitations, our findings are generally consistent between cities and with previously published work conducted elsewhere. This is the first study of its kind among MSM in Mozambique, and provides an insightful snapshot of the relationship between HIV testing and characteristics of MSM. Our study highlights the fact that there is an urgent need to scale-up structural and behavioral interventions among MSM in Mozambique. This scale-up can play an important role in increasing uptake of HIV testing and have a positive impact on curbing the growth of the HIV epidemic.

## Electronic supplementary material

Below is the link to the electronic supplementary material.

Supplementary material 1 (EPS 241 kb)
